# Low-Cost Biobased Coatings for AM60 Magnesium Alloys for Food Contact and Harsh Environment Applications

**DOI:** 10.3390/ijms22094915

**Published:** 2021-05-06

**Authors:** Beatrice Mangolini, Mattia Lopresti, Eleonora Conterosito, Giuseppe Rombolà, Luca Palin, Valentina Gianotti, Marco Milanesio

**Affiliations:** 1 Dipartimento di Scienze e Innovazione Tecnologica, Università del Piemonte Orientale, Via Michel 11, 28100 Alessandria, Italy; beatrice.mangolini@uniupo.it (B.M.); mattia.lopresti@uniupo.it (M.L.); giuseppe.rombola@uniupo.it (G.R.); luca.palin@uniupo.it (L.P.); Valentina.gianotti@uniupo.it (V.G.); marco.milanesio@uniupo.it (M.M.); 2 Nova Res s.r.l., Via D. Bello 3, 28100 Novara, Italy

**Keywords:** magnesium alloy, AM60, biobased coating, PA11, silicon-based coating, polyester lacquer, food contact, chemically aggressive environment, specific migration

## Abstract

Low-cost, environmentally friendly and easily applicable coating for Mg alloys, able to resist in real world conditions, are studied. Coatings already used for other metals (aluminum, steel) and never tested on Mg alloy for its different surface and reactivity were deposited on AM60 magnesium alloys to facilitate their technological applications, also in presence of chemically aggressive conditions. A biobased PA11 powder coating was compared to synthetic silicon-based and polyester coatings, producing lab scale samples, probed by drop deposition tests and dipping in increasingly aggressive, salty, basic and acid solutions, at RT and at higher temperatures. Coatings were analyzed by SEM/EDX to assess their morphology and compositions, by optical and IR-ATR microscopy analyses, before and after the drop tests. Migration analyses from the samples were performed by immersion tests using food simulants followed by ICP-OES analysis of the recovered simulant to explore applications also in the food contact field. A 30 μm thick white lacquer and a 120 μm PA11 coating resulted the best solutions. The thinner siliconic and lacquer coatings, appearing brittle and thin in the SEM analysis, failed some drop and/or dipping test, with damages especially at the edges. The larger thickness is thus the unique solution for edgy or pointy samples. Finally, coffee cups in AM60 alloy were produced, as real word prototypes, with the best performing coatings and tested for both migration by dipping, simulating also real world aging (2 h in acetic acid at 70° and 24 h in hot coffee at 60 °C): PA11 resulted stable in all the tests and no migration of toxic metals was observed, resulting a promising candidate for many real world application in chemically aggressive environments and also food and beverage related applications.

## 1. Introduction

Magnesium alloys have huge potential uses in food contact and general industry applications, because of their low density and high strength-to-weight ratio. In fact, magnesium is the lightest metal used in structural applications, with a density of 1.74 g/cm^3^ which makes it 35% lighter than aluminum and close to polymeric materials, which have a density ranging from the ∼0.90 g/cm^3^ of PP to the ∼2.00 g/cm^3^ of PTFE. Magnesium alloys show shock absorption and damping properties and the melting temperature of 650 °C makes them suitable for casting and die-casting [1]. Production by machining is also possible with a wide variety of possible applications. Its mechanical properties, paired with lightness, make magnesium an interesting subject of research and development in the automotive and aerospace industries where ${\mathrm{CO}}_{2}$ emissions can be contained by vehicles unloading and subsequent fuel savings [1,2]. Wide applications of magnesium alloys are forecast as an answer to the request of reducing emissions for the transport of people and products. One of the major drawbacks of magnesium, being an alkaline earth metal, is its reactivity with water and acids, which can produce hydrogen gas because of Mg oxidation [1]. This reactivity is increased significantly when magnesium is in powder form, and shavings produced during mechanical processing need to be handled with precautions [1]. Today, Mg alloys machining problems were faced successfully and tools and procedures are available. Conversely, corrosion is still a limiting factor to the possible applications despite the attempts in its prevention. Differently from Al alloys, the quasi-passive hydroxide film on the exposed surface is not stable and galvanic corrosion often occurs due to other elements in the alloy as alligands or impurities. To obtain more durable magnesium alloy objects, there are at least three aspects that can be improved [3] (i) the alloy composition, (ii) the exposed surface, (iii) the design of the piece itself. Regarding the alloy composition, the presence of particular elements can be harmful because they can promote the beginning of corrosion. Iron, nickel and copper are the most dangerous impurities for magnesium as they cause galvanic corrosion. Conversely, some other metals (Al, Zn, Mn…) provide protection because of the formation of stable surface phases. The most commonly used magnesium alloys are the AM and AZ types, both have aluminum as main solute, then the first has manganese and the second has zinc and manganese as the other main solutes [3,4]. The use of rare earths or also lithium in the alloys leads to peculiar materials whose use is limited to the aerospace industry due to the higher cost.

It is common use to identify the most suitable alloy for each specific purpose (application and cost), hence proceeding considering the two other aspects (surface exposition and design) which are closely linked. Macroscopic grooves and pores due to design (examples are screws or low-angle bent particulars) are the preferred places where corrosive attacks could occur. Such sites can store the corrosion products which have an auto-catalytic effect on corrosion progress [3,5] and are preferably avoided by means of the piece’s shape forming. The shape is thus optimized by design to avoid such issues, but they cannot be completely eliminated in real world applications. When Mg alloys must be coupled with other metals such as copper, corrosion phenomena are inevitable. After optimizing the alloy composition and shape, the surface exposed to the aggressive environment can be smoothed to hinder the corrosion start by limiting possible corrosion sites [6]. Although these precautions are generally effective for corrosion prevention for most alloys in variable applications, magnesium alloys require further protection with coatings. This is particularly true in outdoor environments and/or in contact with many products, food included. Coatings have the scope of preventing the contact between the alloy and the oxidants, and must be effective on planar but especially on edgy or pointy parts of the artifact.

The optimal coating must be, on one hand, cost effective, with easy applicability and low environmental impact and, on the other hand, able to protect artifacts in a chemically aggressive environment regardless of the sharp edges and the corrosion susceptibility of the alloy. A coating fulfilling these conditions would allow a wider diffusion of magnesium alloys both in general industry and in the food contact field [7]. There are many types of treatments, substances and materials which are promising for protecting the alloy depending on the environment of application. Cost, easiness of applicability, performance and environmental impact depend of course on the chosen strategy and the best solution can be a balance of these four aspects. To be exploited in general industry and/or food industry, they must meet cost, scalability and low toxicity requirements other than providing corrosion protection. For instance, electrophoretic deposition of graphene oxide [8] allows an optimal protection but it is very expensive, and it also requires polluting reactants like concentrated sulfuric acid and NaOH 5M in the preparation. Laser surface melting [6], despite very efficient, is an energy and time-consuming process. Two strategies, based on vacuum or ambient surface treatment, can be envisaged for high level applications: the formation of stable oxide layers on the surface with further application of paints and enamels or plasma enhanced processes, such as deposition of hard and dense materials, or strong oxidation of the surface. High Velocity Oxygen Fuel (HVOF) is one of the thermal spray techniques that have shown an enormous potential for surface modification of different metals. The high particle velocities and moderate temperatures achieved by HVOF drew a good academic interest towards Mg alloys treatment, as they lead to very dense coatings with outstanding wear behavior and superior bond strengths [9,10]. The Plasma Electrolytic Oxidation (PEO) [11] method is environmentally friendly, and the treatment is normally performed in aqueous solutions without the addition of toxic compounds. The formation of PEO coatings is possible on almost all cast and wrought magnesium alloys [12,13], with the result of dense coating structures and good corrosion resistance. These methods, despite very performing, are costly and hardly applicable on the wide scale required at industry and commercial levels, where powder or spray coating are widely employed.

With the aim or pursuing low-cost, environmentally friendly, biocompatible and easily up-scalable solutions for the protection of the lightweight alloy, AM60 magnesium planar edgy test samples and then artifacts were coated exploiting three different approaches. A powder coating (polyamide 11, from now on PA11), commercially known as Rilsan^®^ [14] was chosen, as its use is well established on iron-based alloys especially concerning pipelines [15]. PA11 has the advantage of being biobased, it is obtained from renewable resources. e.g., from castor oil, thus exploiting poor and semi-arid soils not suitable for food cultivars. It is very versatile, due to the possibilities for its functionalization with silanes [16] or for blending with poly(butadiene succinate) (PBS) to obtain a partially biodegradable matrix [17]. In addition, PA11 exhibit antibacterial properties as recently demonstrated [18]. These examples support good features which allow the tailoring of the coating according to specific requests.

Two synthetic coating families, one silicon-based and the other consisting of polyester lacquers, typically used for Al alloys, were tested as a comparison. These three kinds of coatings are commonly used in the protection of iron [14] and aluminum alloys [19,20] which are among the most widespread alloys but, to the best of our knowledge, they were never tested on magnesium ones, probably for the different reactivity of its surface, and for the very limited applications in general industry.

Therefore, the scope of this work is evaluating if these coatings can be repurposed for the application of different materials such as Mg alloys (with different surface reactivity with respect to traditional substrates of these coatings), and which is the best solution in terms of both protection and facility of scaling-up toward industrial applications Planar edgy samples and, finally, prototype artifacts were tested in increasingly aggressive environments (i.e., solutions with increasingly acidic or basic nature and salinity, to simulate their use in harsh conditions such as automotive field during de-icing or marine environment, and contact with aggressive chemicals or foods) and particular attention was dedicated to resistance in contact with food and cleaning solutions, at room and higher temperatures to simulate real world usage. Migration studies have been carried out by immersion of the sample as indicated by UNI EN 1186-3:2002 [21] for plastics and the simulants were analyzed by ICP-OES. Optical and ATR-IR microscopies were used to assess the effects of chemicals and simulants on the coatings. These two methods allowed a non-destructive analysis of the samples before and after chemical tests, contrary to Electron microscopy (SEM/EDS) analysis of corrosion products, which required the samples to be cut to fit inside the vacuum chamber. SEM/EDS was thus used for textural observation of the coatings (cut from samples) and for element analysis assessment to investigate coating and additional features.

## 2. Results

**Figure 1:**
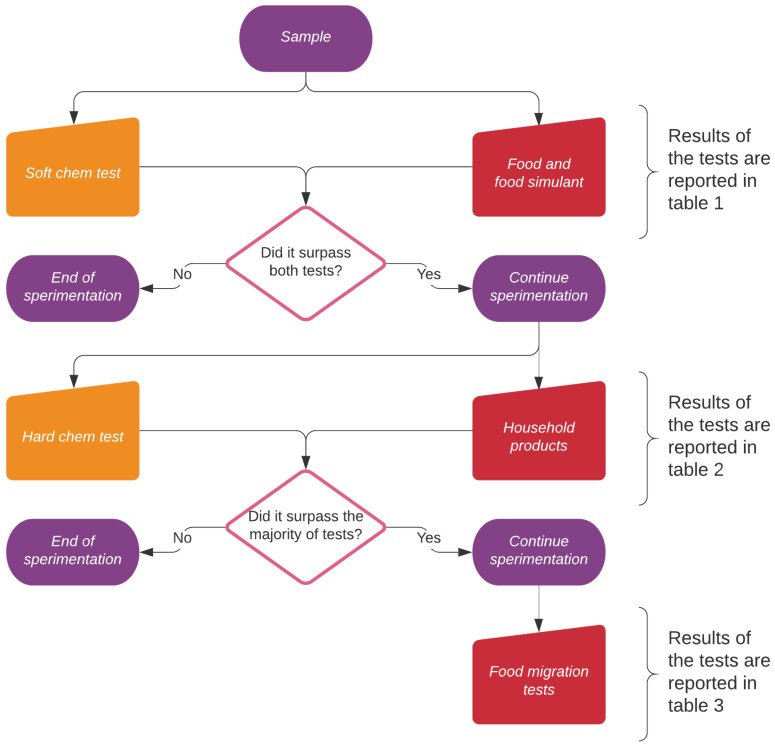
The samples were tested in three levels of increasing aggressivity. The first level is composed of two mild drop tests (one chemical and one with food and food simulants). The second level includes more aggressive agents, including harsh cleaning products. The last level concerns immersion migration tests on samples and on prototypes of coffee cups.

The performance of the biobased PA11 coating on AM60 alloy were compared to organic lacquers and silicon-based coatings. Several samples were produced, and a variety of test were carried out to cover both chemically aggressive environments and food contact applications, as detailed in the experimental section. The experimental strategy is summarized in Figure 1 and it is divided in three levels:

1. all the samples initially faced soft chemical and food drop test to carry out a first selection (first level of Figure 1), and the results are summarized in Table 1;
2. the samples passing the first tests were subjected to hard chemical aggression and contact with real world household products (second level of Figure 1) and the results are given in Table 2, Section 2.1.1. Drop tests using aggressive solutions of strong acids, bases and salts were carried out at increasing concentrations and temperatures to assess possible usages in harsh environments and to simulate aging during long term usage. These tests allowed to understand the intrinsic shape-independent resistance of each coating;
3. the suitability for food contact applications of the best performing samples was then tested using the food simulant solutions described in the UNI EN 1186-3:2002 norm (third level of Figure 1 and Table 3); finally, immersion tests were carried out (also using real world prototypes, e.g., coffee cups) to measure, by ICP-OES, the amount of metals released and to calculate the specific migration. Dipping tests allowed to introduce the shape effect, since edgy parts are very critical in favoring corrosion.

Real world prototypes in the form of coffee cups were produced using the AM60 alloy to assess the effects of size and shape in the migration tests carried out just by filling the cup in real use conditions. Prototypes were tested in parallel with standard flat laboratory samples.

### 2.1. Drop Tests

Drop test were carried out by deposing a drop of solution on a flat sample. This approach allows the efficient investigation of the intrinsic shape-independent resistance of the coating and the effect of repeated contact within time as detailed in Section 5.2). Moreover, it allowed the inspection of the damages by optical and ATR-IR analysis.

#### 2.1.1. Chemical Aggression Tests

The samples listed in Table 1 underwent drop tests to obtain a first screening with respect to their chemical resistance. The GSF organic lacquers, after the failure of some of the first tests, were prepared again increasing the thickness of the coating. For this reason, the thinner (12 μm to 13 μm) GSF coatings were not subjected to soft chemical aggression tests since they failed the acetic acid 10% test. To undergo food simulant tests, thicker (30 μm to 40 μm) versions of the same coatings (both transparent and white) were prepared, and passed the same tests. The drop tests with saline, acidic and basic solutions (described in the materials and methods Section 5.2) were performed by increasing the concentration of the solutions and then introducing the temperature effect.

##### Soft Chemicals

**Figure 2:**
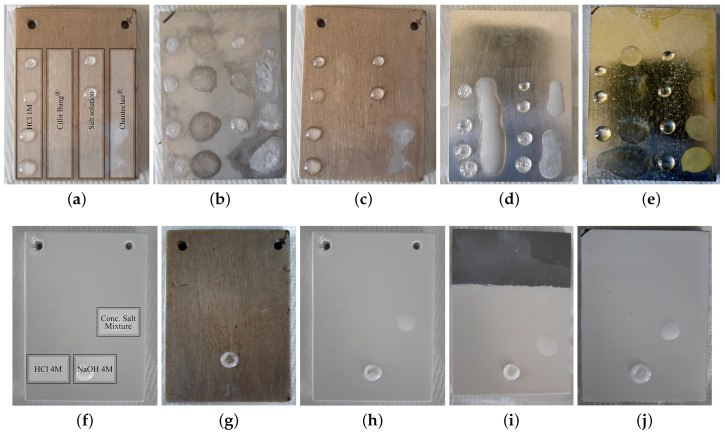
Selected planar samples at different stages of RT (top) and high temperature (bottom) drop tests: (**a**,**f**) show the scheme of the solutions used for the drop tests; (**b**) bare magnesium shown as a reference; (**c**–**e**) are transparent PA11, transparent nanoceramic and polyester coated samples respectively; (**g**,**h**) are transparent and white PA11 coated samples; (**i**) is the white siliconic coated sample; (**j**) is the white GSF lacquer 40 μm coated sample. Bottom pictures are taken after the high temperature test in the oven, and the NaOH pellet formed during drop evaporation is evident.

A HCl 1M solution and a salt mixture with both NaCl 5% *m*/*v* and CaCl_2_ 5% *m*/*v* were used. The test was performed adding a drop every hour as detailed in Section 5.3.1. After five hours, the samples were pat dried and examined under the optical microscope. Surface alterations were visible on the bare magnesium (Figure 2b) while signs of reaction (bubbles) could be seen on the transparent nanoceramic coated sample (Figure 2d). The transparent polyester lacquer sample (Figure 2e) was uneven on the edges, suggesting bad adherence to the substrate. Thus, these three samples were not subjected to more aggressive tests. The other samples, like the transparent PA11 (Figure 2c), passed the test, as summarized in Table 1 and underwent harsh solution and higher temperature tests. Samples failing the chemical aggression tests were still tested for food applications, since the conditions are usually milder. These data are detailed in Section 2.2, where all food contact tests are grouped.

##### Harsh Solutions and Household Products

In the perspective of household and office use, as real world aggressive test, two commercial cleaning products (a degreaser and a descaler) were then tested. The products were chosen to be opposite in the pH scale, in fact the descaler Cillit Bang^®^ is acid (pH =0.8) and the degreaser Chanteclair^®^ is basic (pH =11.5). Due to the surfactants inside these commercial products, some drops joined together as it is clearly visible in Figure 2d. PA11 samples are much less wettable by polar solutions as can be seen by the drop shape in Figure 2c with respect to Figure 2d,e. Only GSF transparent lacquer failed these tests.

Three solutions were used on the samples which passed the tests in Table 1: HCl 4M, NaOH 4M and a concentrated salt mixture with both NaCl 10% *m*/*v* and CaCl_2_ 10% *m*/*v*. One drop of each solution with a volume of 30 μL, was deposited every hour, and after the third hour, the samples were rinsed and dried. The samples were not visibly damaged in this case. Then, the same test was repeated using a single drop of 40 μL (to limit evaporation) for each solution but the samples were in a muffle oven at 50 °C for 1 h. The results are summarized in Table 2. The PA11 and the GSF lacquers coated samples resisted to this last testing step as it is shown for the transparent and white versions in Figure 2g,h. The white siliconic and the white GSF lacquer 40 μm coatings were slightly damaged by the hot NaOH solution that left a stain where the drops were deposed. The samples are shown in Figure 2i,j but the staining is not easily appreciable from the pictures.

#### 2.1.2. Optical Microscopy Analysis

**Figure 3:**
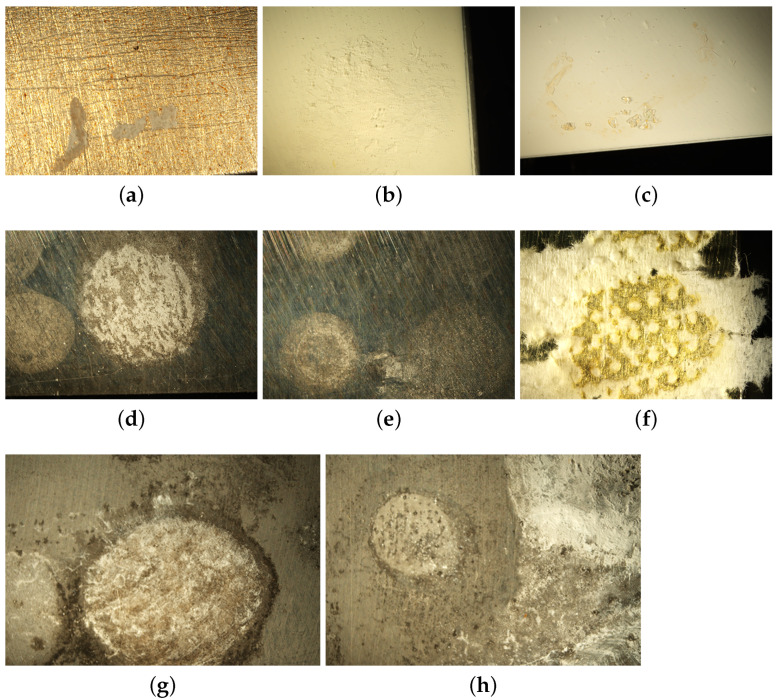
Optical Microscope images of selected samples at 6.5× magnification. Salt residues deposed on transparent PA11 transparent coating (**a**) and on white GSF lacquer (**b**,**c**); damaged spots on transparent nanoceramic Easysol (**d**,**e**); damaged spots on transparent GSF lacquer (**f**); corrosion signs on the bare magnesium sample (**g**,**h**).

After the drop tests, the samples were examined under the optical microscope to find possible signs of corrosion or damages in the coatings. In Figure 3 some pictures of damaged spots are reported. The commercial cleaning products left some residues which appeared like tiny crystals or stains. When the coating underneath was not damaged, the residues were eliminated by scratching or washing with deionized water. These residues were found on the transparent PA11 coated samples and on the GSF white lacquer coated sample as shown in Figure 3a–c. This and all other PA11 samples resulted inert to the test and all residues were easily removed by simply rinsing with water. On both the GSF transparent lacquer coated samples, a reaction occurred as the texture changed in the spots attacked by the liquids. There, the coating became sticky, and the paper used to dry it remained stuck on the spot as it can be seen in Figure 3f. The white GSF sample passed the test without damages, thanks to its increased thickness (evident by the cross-section SEM image reported in Figure S1 of the supplementary information file) and/or the protective effect of the inorganic additive inserted to obtain the white color. The Nanoceramic Easysol coated sample was damaged by all four solutions employed in the test as shown in Figure 3d,e. The white and brown siliconic coated samples were not damaged and no residue was left on their surfaces, hence they are not shown. As expected, the bare magnesium was corroded by all the solutions and the affected spots are clearly visible in Figure 3g,h.

#### 2.1.3. Infrared Microscopy Analysis

**Figure 4:**
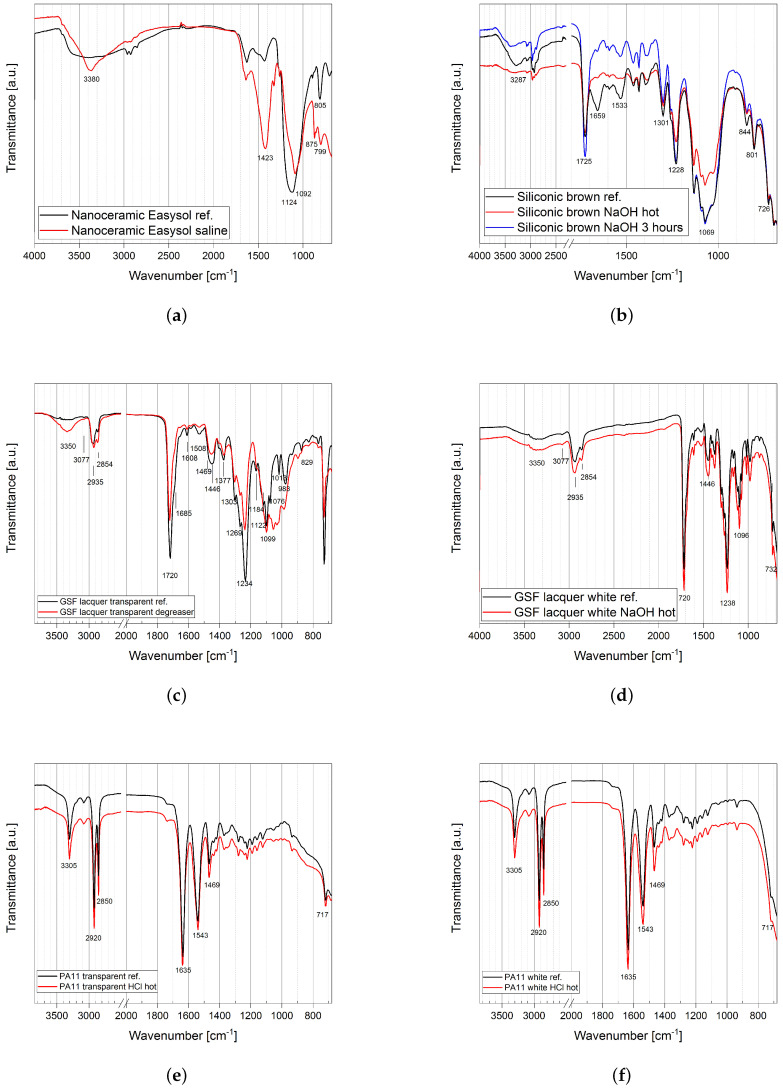
ATR spectra of sample before and after contact with chemicals at different levels of aggressivity to highlight corrosion or inertness of the coatings: (**a**) damages on the transparent nanoceramic coating by the saline solution; (**b**) brown siliconic coating showing slightly different spectra; (**c**,**d**) transparent and white thick GSF lacquers showing that the basic degreaser corroded the transparent coating but not the white one; (**e**,**f**) transparent and white PA11 samples showing no difference between the spectra of the reference and of the spots in contact with hot 4M HCl solution.

Infrared spectroscopy was performed in ATR mode. Each sample was tested in each spot that was altered after initial the drop test and, as a reference, on a uniform and regular area where the coating was untouched (top part of the sample or its rear side). The spectra of the three classes of coatings before and after the attacks are reported in Figure 4 and the position of the main bands in each sample summarized in Table S4 in the supplementary information file. The transparent nanoceramic sample is silicon-based and its spectrum in Figure 4a is similar to those of the two siliconic samples in Figure 4b. In particular the bands around 1092 cm^−1^ is due to the Si−O−Si asymmetric stretching while the band at 1423 cm^−1^ is related to $\mathrm{Si}-{\mathrm{CH}}_{3}$ groups deformation. The nanoceramic coating is very thin and the spectra acquisition suffered from this, leading to non-sharp peaks, hard to be identified. However, the difference between the reference and the spot where the drop of saline solution was deposed is clear: the saline solution, after four hours of contact, chemically modified the coating deeply and permanently, already at room temperature. White and brown siliconic samples have characteristic signals similar to the nanoceramic due to the presence of silicon atoms. The FTIR-ATR spectra of the brown sample before (black line) and after the aggression by NaOH at room temperature (blue line) and at 50 °C (red line) are shown in Figure 4b. The spectra of the white siliconic coating are not shown since they are very similar to those of the brown one, except for the band at 1659 cm^−1^ which is not present in white one. The characteristic bands are the Si−OH stretching around 844 cm^−1^ and the large multiple band of Si−O−Si asymmetric stretching vibration in the range 1050 cm^−1^ to 1200 cm^−1^. The length of the polymer chain influences the width of the band, which appears divided into three peaks in the region 1100 cm^−1^ to 1000 cm^−1^: the longer the chain, the broader is the peak. The deformation vibration band of $\mathrm{Si}-{\mathrm{CH}}_{2}R$ groups is located at 1228 cm^−1^ and it is sensible to the chain length, decreasing in intensity as the length of the aliphatic chain increases. The spectra collected in the spot in contact with hot sodium hydroxide solution reveal different intensities with respect to the reference spectra. By looking at the spectra, it can be seen that the most intense band at 1069 cm^−1^ decreases in intensity while other peaks change in shape (at about 1533 cm^−1^ and 1659 cm^−1^) meaning that both brown and white coatings were damaged. Moreover, in the spot after contact with hot NaOH the color of the brown siliconic sample was visibly faded while in the white siliconic sample there were no visible changes. The GSF lacquers coatings are resins of polyester *tere*- and *iso*-phthalic acids. The main features in their FTIR spectra (shown in Figure 4c,d) are the strong band at 1720 cm^−1^, typical of C=O stretchings of esther groups while the band at 1234 cm^−1^ is due to the C−O−C asymmetric stretching of saturated aliphatic esthers. The band at 2935 cm^−1^ and 2854 cm^−1^ is due to aliphatic CH stretching vibrations. The wide band centered at 3350 cm^−1^ in the white GSF coating (Figure 4d) is attributable to OH stretchings due to the white $\mathrm{Ti}O_{2}$ pigment as well as the bands under 800 cm^−1^. The transparent GSF coating was heavily damaged by the contact with the basic degreaser. The spectra reported in Figure 4c show in particular the appearance of new signals in the coating damaged by the degreaser in the region around 3350 cm^−1^ that can be related to NH and OH stretchings of an ethanolammine residue from the product as well as the new bands in the region between 1080 cm^−1^ to 1000 cm^−1^. The spectra of the white GSF lacquer, after the test with hot HCl, shown in Figure 2j, are reported in Figure 4d and do not show any sign of degradation from the test. Since their compositions are identical except the inorganic additive used to obtain the white color (demonstrated by SEM/EDX analysis), the protective effect in the white GSF lacquer version can be ascribed to its increased thickness and/or to the presence the titanium oxide in its formulation. The spectra of the PA11 coatings are shown in Figure 4e,f and have few characteristic bands. At 3305 cm^−1^ falls the NH group stretching while the bending for the same group is found at 1543 cm^−1^. Asymmetrical and symmetrical stretching bands involving ${\mathrm{CH}}_{2}$ groups are visible at 2920 cm^−1^ and 2850 cm^−1^ respectively. The CO stretching band is located at 1635 cm^−1^ while the ${\mathrm{CH}}_{2}$ bending falls at 1469 cm^−1^. The out-of-plane bending for the same group is at lower wave numbers (717 cm^−1^). The typical PA11 coating bands and signals were detected unchanged after all the drop tests confirming that the coating was not damaged, also at the chemical level. In Figure 4e,f, the reference spectra of transparent and white PA11 coatings are shown together with those measured in the hot HCl spot, i.e., the more aggressive situation for a magnesium alloy. Visual inspection suggested a faint change in the surface appearance, looking opaquer than before contact, but the spectra do not show any variation suggesting only minor modifications and thus very good resistance also to hot acid attack.

### 2.2. Food Contact Tests

To the present date, the principles of safety and inertness for all Food Contact Materials are regulated in the EU by the Commission Regulation (EC) No 1935/2004 for specific types of materials such as plastics, while the regulation for other materials are adopted locally by each EU Member State in accordance with Article 6 of Regulation 1935/2004. In Italy the characteristics of stainless steel for food contact are dictated by D.M. 21 marzo 1973 and subsequent amendments; and D.P.R. 777/1982. The coatings used in this study are commercial and compliant to the EU regulations about plastic materials, but our interest is to determine if there is migration from the underlying magnesium alloy, therefore we devised a procedure to evaluate the specific migration based on the existent legislation.

Food contact tests are milder than the test in the previous section. For this reason, also the samples failing some harsh chemical tests were considered in the experimentation, in view of possible applications in less aggressive food contact environments (neutral foods and drinks, greasy and oily substances). Also in this case, the first selection is done by the drop tests, while the more promising samples underwent migration tests also on real world prototypes. The contact with food did not usually leave visible marks on the surface of the coating but the reaction with acidic solutions was demonstrated by bubbles indicating the aggression of the alloy with the reaction:

{1} $${\mathrm{Mg}}_{\left(s\right)}+{2H_{3}O}_{\left(l\right)}^{+}\to {\mathrm{Mg}(\mathrm{OH})}_{2(s)}↓+{2H}_{2(g)}↑$$

#### 2.2.1. Food Contact Drop Tests

**Figure 5:**
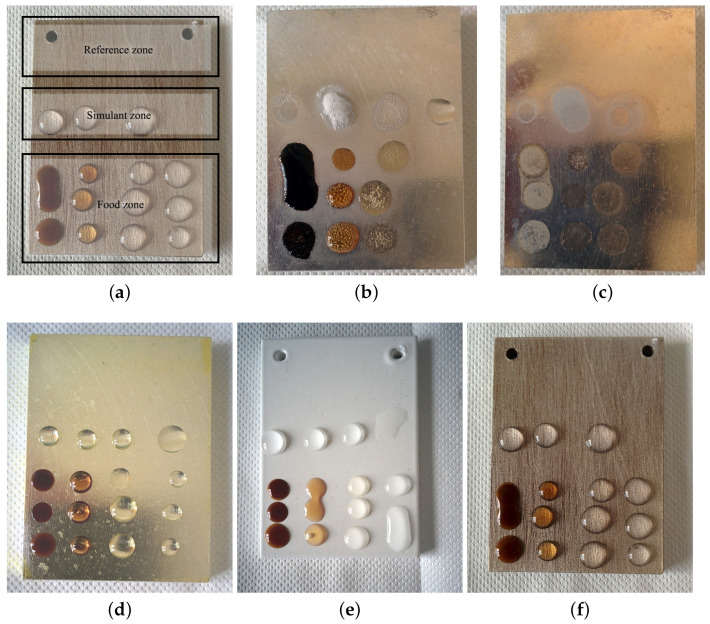
Drop test on uncoated AM60 alloy (top) and (**d**) GSF lacquer, (**e**) white and (**f**) transparent PA11 coatings (bottom); (**a**) shows how samples were divided for the different analyses: In simulant zone the four drops are A, B, C, D solutions; In food zone, from left to right coffee, Coca Cola^®^, white sparkling wine and 10% acetic acid. (**b**,**c**) show the bare magnesium sample during the test and after rinsing. (**d**–**f**) show GSF transparent lacquer 12 μm, white PA11 and transparent PA11 transparent respectively.

Four samples were used for this test: the first one was not coated bare AM60 as reference, one was covered with the transparent lacquer coating, one treated with a primer and then coated with white PA11, one treated with a primer and then covered with transparent PA11. The first test was done placing a drop each of coffee, Coca Cola^®^ and white sparkling wine every hour and refilling the previous drops every hour to obtain different contact times with the solutions. On another spot of the sample, drops of the simulants A, B, C, D and 10% acetic acid were deposed on the surface and left there for 4 h proceeding as in the previous test. Although the surface of the unprotected AM60 alloy was noticeably damaged by the contact with all solutions, as also demonstrated by the bubbles, due to hydrogen gas formation, during the test, the coated samples did not show any trace of damage after removing the drops, as shown in Figure 5. The different wettability of the surface is evident by looking at the shapes of the drops of these polar water-based foods: on white and transparent PA11 the drops are well formed and raise from the surface with a higher contact angle, indicating a hydrophobic less wettable surface. On bare magnesium (Figure 5b) the drops are flat and widened because of its polar surface and higher wettability by polar solutions. GSF lacquer (Figure 5d) has intermediate wettability between the bare alloy and PA11 samples (Figure 5e,f).

#### 2.2.2. Migration Tests by Dipping and Elemental ICP-OES Analysis

**Figure 6:**
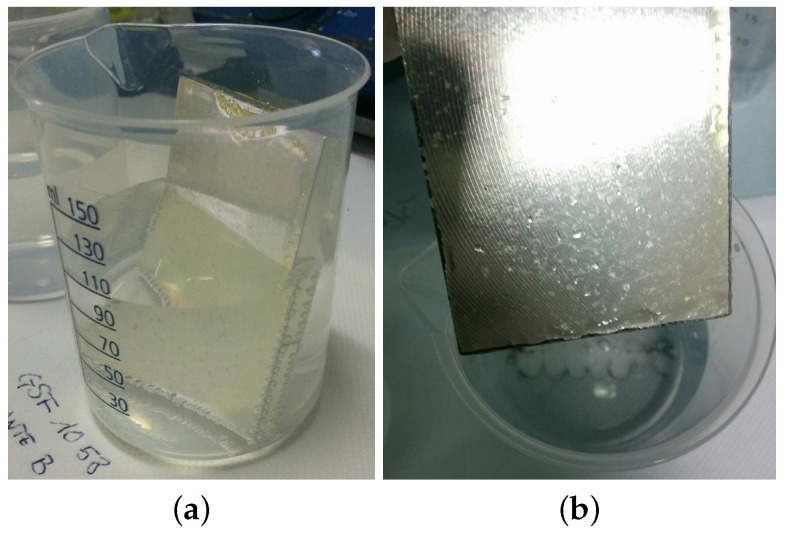
Dipping test on AM60 alloy with lacquer GSF coating during (**a**) and after (**b**) the test.

The samples were tested by immersion into the simulant solutions to determine the specific migration from the samples. To perform this test, simulant B, i.e., the most aggressive solution for the alloy among the four suggested by the norm, was chosen. Each beaker was filled with 100 mL of solution B and the samples were dipped in the solution for 24 h at RT. A similar beaker containing only 100 mL of solution B was left in the same conditions to be used as a reference. The simulants were then analyzed by ICP-OES to assess both the presence of the metals that are known to be part of the alloy and of other metals that pose health risks, the aim being on one hand to assess the corrosion protection given by the coating and on the other hand the safety of use. The lacquer GSF 30 μm coating showed the formation of evident bubbles (see Figure 6a) on the edges of the sample. The test was stopped after about 2 h after observing the formation of a dark precipitate in the solution and noticeable corrosion of the edges (see Figure 6b). Despite performing better than the with GSF 40 μm sample, the white siliconic coating proved to not be suited to protect the sharp edges of the standard planar edgy sample and signs of corrosion were observed during the dipping test. Therefore, a new sample with rounded edges was produced and coated with the siliconic brown coating and used for all subsequent tests. Also in this case, despite the stability increased, small bubbles were noticed on the edges, indicating the aggression of the alloy. The specific migration of Mg across the siliconic brown coating resulted in being 1.2(4) mg/dm^2^. The complete set of data are reported in Table S1. The use of this siliconic coating is therefore limited by the geometry of the sample when a uniform and thick coating is difficult to achieve such as in the case of sharp parts and/or irregular surfaces.

When testing the PA11 samples, the formation of bubbles was not detected and there were no visible signs of corrosion neither in the white nor in the transparent sample. The ICP-OES measurements did not reveal migration of Mg or other metals from the sample in the simulant B used in the contact. The specific migration for each element calculated from these tests is available in Table S2 in the Supplementary Materials.

The thickness of the coating proved to be a critical factor to prevent corrosion especially when the object has sharp edges, on which the coatings tend to be thinner, and imperfections are more frequent.

**Figure 7:**
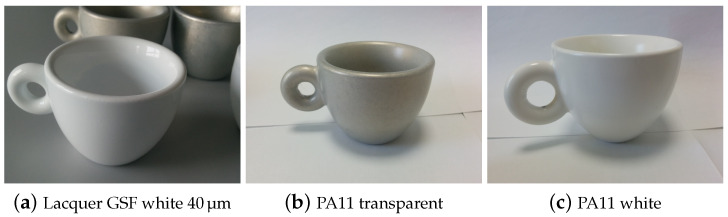
Prototypes of coffee cups made in AM60 magnesium alloy with different coatings.

The set of tests on prototypes (Figure 7) was carried out on the best performing coatings from the first and second levels drop tests as in Figure 1. Transparent and white PA, the white siliconic coating and the thicker GSF white lacquer 40 μm were tested using simulant A and B at 20 °C for 24 h and at 70 °C for 2 h. Simulant C (10% ethanol) was only tested at 20 °C for 24 h. The cups were filled with 40 mL (small coffee cups,) and 120 mL (large coffee cups,) of simulant. The surface to volume ratios were 1.23 and 0.95, respectively. ICP-OES analysis on the recovered simulants revealed traces of metals in the simulant B that was in contact with the white siliconic and the white GSF lacquer coating for 2 h at 70 °C (Table 3 and Table S2). The specific migration of Mg resulted in being 0.09(5) mg/dm^2^ for the GSF lacquer and 0.70(2) mg/dm^2^ for the siliconic coating. The migration of Mg is not limited by the regulations since it does not cause concerns on health, therefore we evaluated it as an indicator of the amount of corrosion. When the corrosion is significant, traces of Al (a known bio-active and possibly harmful element) are also found in the solution, being the main alloying element. Also in this test, the PA coatings proved to be effective in protecting the underlying alloy since no significant amount of magnesium is released from any of the samples coated with PA11. Most releases of toxic metals were below the LOD or, if significant, very small and below the norm values (Table S3). All the results and the calculated specific migration from these samples are reported in the supplementary information file in Table S2. At last, a real world usage was simulated by an “aging coffee test”, carried out on the prototypes with PA11 transparent and white coatings, siliconic white coating and with GSF white lacquer using hot coffee. The cups were filled with coffee produced by an Italian Moka and kept continuously in an oven at 60 °C for 24 h to simulate aging. At the end of the test the cups were emptied and washed with water and soap. The result was similar for the PA11 coatings and white GSF lacquer that were stained at the end of the test but kept a perfect adhesion. The mild staining in such conditions of continuous contact with hot coffee should not be a problem in real world conditions where usage is alternated to cleaning. However, to fully pass the “aging coffee test” without any staining of PA11, the only solution is the deposition of an additional inorganic coating (a preliminary successful test was made by silica deposition by CVD), but it is beyond the scopes of the present work, i.e., exploring low-cost and easily scalable process. The siliconic coating did not pass the “aging coffee test” because even if it was not stained a severe detachment was observed.

## 3. Discussion

The results of the tests and characterizations are discussed in light of the compositional/morphological features of the samples, obtained by SEM/EDX, using literature data as reference. The bare alloy showed moderate reactivity with real drinks and food simulant (Figure 5b,c) suggesting its intrinsic resistance, without any coating [5]. Moreover, also in the presence of corrosion and coating failure during migration tests, a reduced metal migration with the absence of toxic metals was observed, thanks to the AM60 alloy composition and purity: this aspect allows envisaging larger applications of AM60 in food contact and all those fields where low releases of toxic metals are mandatory, as, for instance, medical and dentistry fields. The three investigated classes of coatings showed good to optimal responses when drop tests are carried out, with none or very limited corrosion (Figure 5d–f). The drop test approach resulted very time-saving and gave preliminary indications about the specific resistance of the coating to chemicals and simulants, and, at the same time, allowed an efficient study of the damages by optical and ATR-IR microscopies. Drop tests allowed (i) to exclude the coatings with very poor performance, (ii) to check if preliminary treatments such as tumbling and sandblasting influence the performance of the coating, (iii) to assess that the effects of the primer under the PA11 coating are negligible.

The main limitation of drop test resides in neglecting the shape effects, since edgy and pointy part of the sample are not tested. Migration tests by total immersion of planar samples are complementary to the drop test and they highlighted the limitation of the thinner GSF and silicon-based coatings (10 μm to 20 μm), failing these tests due to the insufficient thickness on the edges and presence of defects. Since the siliconic coating is already rather thick, smoothed edges samples were tested showing limited yet present corrosion, thus suggesting restricted applications of silicon-based coatings and GSF lacquers only on rounded artifacts. The combination of drop and dipping tests allowed selecting the best coating for real world prototypes, e.g., small and large coffee cups (Figure 7). Interestingly, the white version of the organic lacquer (GSF white 40 μm, obtained by adding a titania-based pigment in about 15% weight, to transparent GSF recipe) performed much better in real world tests with hot food. This result can be ascribed to the protective effect of the inorganic additives (CaCO_3_ and TiO_2_ as indicated by SEM/EDX), added for aesthetic purposes. As envisaged by Rong-Gang Hu et al. [7] powder coating allows the preparation of uniform coatings of high thickness that proved to be the most effective in protecting the underlying AM60 alloy and are also environmentally friendly since they do not require the use of polluting solvents. The application of PA11 on AM60 alloy proved to be feasible and the performance of this coating were similar to those obtained when deposed on steel [15]. Biobased PA11, in both transparent and white versions, resulted inert to concentrated acid and basic solutions at room and higher temperature. They resisted also to concentrated salt solutions common in food and marine environments (NaCl) and in the presence of de-icing agents (CaCl_2_), very important for automotive industry applications. These performances, despite known on steel [22], were demonstrated for the first time on Mg alloys, showing completely different surface features and reactivity. Moreover, the white PA11 also allows the use of primers to further improve adhesion, without modification of the color, occurring when using a transparent PA11 with a colored primer.

Overall migration tests, performed on the selected PA, organic and silicon-based coatings, allowed to assess their suitability for food industry, where it is important to prevent any migration of metals in the food. ICP-OES measurements reported in the Supplementary materials showed that the best performing coatings are the PA11 ones. Conversely, the analyses on other coatings highlighted signs of permeability, allowing the substrate to be attacked by the simulant solution and therefore releasing Mg and Al.

**Figure 8:**
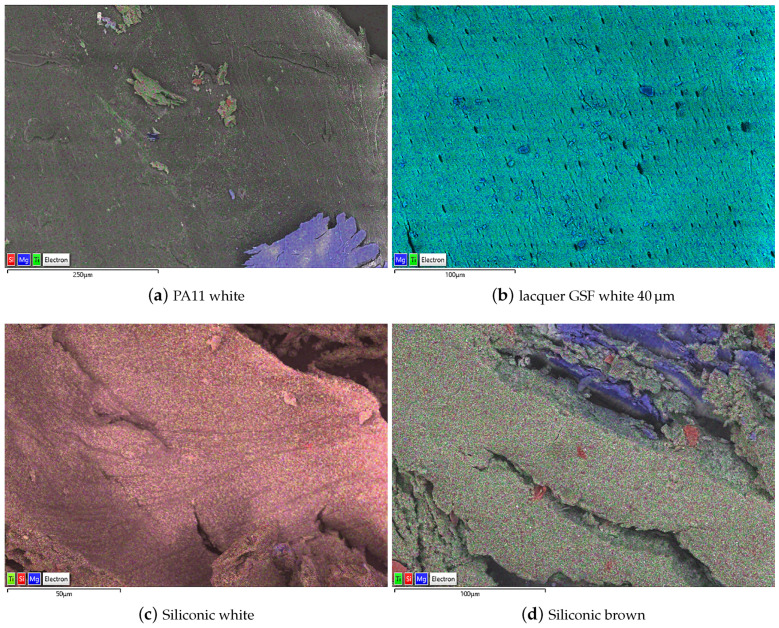
EDS maps of the four samples of the third step of analysis.

The SEM micrographs of Figure 8, superimposed to EDX elemental analysis maps allowed explaining the results here above discussed. The PA11 coating (Figure 8a) can be cut from the surface as a “bulky fragment” with homogeneous aspect and without pores and defects, also in a large area of about half a mm. In the bottom right corner a slice of AM60 alloy is visible and was cut attached to the PA11 coating. These features explain the very good adhesion of the coating and its optimal barrier to chemicals to provide the best protection. Conversely, the organic polyester lacquer (Figure 8b) is cut in a tiny and still homogeneous slice, but with evidence of some degree of porosity. This morphology assures a good continuity of the coating explaining the good performance when used in the thicker versions. The pores explain the releases observed in the migration tests. The silicon-based coatings (Figure 8c,d) are vitreous and, when cut, they resulted broken in much smaller fragments, requiring a higher magnification to visualize a single piece. Many fractures are present, explaining the permeability to chemicals and the failures in the aggressive and real world test.

## 4. Conclusions

Considering all the tests (drops, dipping, real world prototypes), the biobased PA11 coating is the unique with no failures. PA11, especially in its white version, resulted thus a good candidate for technological applications of various kinds, from harsh environment (salty, acid and basic chemicals up to 70 °C) to food contact applications. Colored versions of the PA11 coating to fulfill aesthetic purposes, can be easily obtained with commercial grades, already exploited in steel coating industry. The combination of an alloy with low impurity levels to a biobased very stable coating allowed obtaining migration levels of toxic metals beyond detection limits, very interesting for many fields (food but also medical/dentistry). Thanks to its thickness, PA11 showed good performance also with edgy shapes. Its low cost and already known wide applications as steel coating suggests an easy industrialization of the proposed coatings also on magnesium alloys. In fact, the PA11 powder coating can also be deposited by dipping the sample in polymer powder fluidized bed. The powders adhere electrostatically and are then fused and polymerized in an oven. The biobased origin of PA11 suggests a lower environmental impact with respect to other coating and the low or absent toxic metal release an optimal level of biocompatibility. Finally, the chance of easily modifying the PA11 properties as in ref. [18], suggests the possibility of tailoring and improving coating properties to specific purposes, such as improved oxygen barrier effects and/or specific bioactivity such as biocide activity and controlled drug release.

## 5. Materials and Methods

### 5.1. Samples

The samples are rectangular pieces 50 mm × 70 mm × 5 mm, with 90° cut edges, if not differently specified, of AM60 magnesium alloy (composition 93.5% magnesium, 6% aluminum, 0.35% manganese and 0.1% zinc), with edgy borders to simulate real world critical shapes. AM60 samples (with this shape or customized) can be purchased by Dongguan Dechuang Hardware Co. (Vanke Center, No. 1, Changqing South Road, Chang ’an Town, Dongguan City, Guangdong Province, China) by Alibaba website [23]. The samples were treated with three classes of coatings. Biobased commercial Polyamide (PA11) coatings called Rilsan^®^ [16], produced by Arkema (Rho, Milano, Italy) was deposited as powder coating, after heating the metallic samples at 200 °C. Four samples were prepared: 2 in a transparent version and 2 in the white one. Coatings were used as purchased and white version contains a standard commercial inorganic pigment as confirmed by SEM/EDS analysis. The samples differ also for the superficial treatments applied to favor the adhesion: one of each color was subjected to tumbling and the other to sandblasting. In a preliminary test, each kind was coated by an epoxy acrylate-based resin solution as primer (commercial name PRIMGREEN LAT 12035) and then powder coated with PA11. However, the primer is slightly yellowish, and it is visible under the transparent PA, with a bad aesthetic effect. Because corrosion performance, in these preliminary tests, was almost independent using the primer, the transparent coatings were applied without the underlying primer. Since the performance of tumbled and sandblasted samples was identical, only sandblasted samples are presented. Then three routes were explored in the family of silicon-based coatings. Nanoceramic Easysol is a transparent coating [24], formulated by polymeric glasses comprising sodium and/or potassium silicate solutions, lithium polysilicate solutions, silanes and water-soluble polysiloxane-based surfactants. Ceramic Techsol is a white coating based on Sol-Gel technology [19], in which hybrid organic/inorganic substances as alkoxides are reacted by heating in water in the presence of a mineral acid and a non-ionic surfactant to obtain a hard and rigid matrix with specific surface properties. The use of an organyl oxysilane species up to 37% in the mixture can increase the hydrophobic behavior of the surface. Siliconic white and brown are polysiloxane-polyester coatings, similar to the Sol-Gel ceramic component but with a more organic character. All silicon-based coatings were produced by SIVE (Cirié, Torino, Italy). The nanoceramic transparent coating was applied by dipping; the sample was then cured at 250 °C. The ceramic coating was obtained by spraying two different layers, where the overlying layer had a hydrophobic behavior. The siliconic coatings in white and light brown color were applied by spraying the samples and then curing them at 280 °C. Standard polyester lacquers (white and transparent with different thicknesses) typically used for aluminum coating for food application were deposited by spray coating and then cured by UV lamp by Geolac S.r.l. (Tortona, Alessandria, Italy) [20]. The thickness of the siliconing coatings and GSF lacquers were defined by (spraying or dipping) machine parameters. Thickness is increasing from nanoceramic and ceramic coatings (5 μm to 10 μm) to siliconic and GSF lacquers (10 μm to 40 μm) to PA11, where the thickness depend on the size of the starting powders (120 μm to 150 μm). To confirm the coatings’ thicknesses, first a profilometer and then the SEM analysis were led, on those samples where a coating slice could be cut in a suitable size for the SEM sample holder. The cross-section of the GSF 40 μm lacquer is reported in Figure S1 of the supplementary information file.

### 5.2. Solutions

Different liquid solutions were used in the tests with different purposes. To test the possibility of using the alloys in increasingly aggressive real world or laboratory environments, a salt mixture with both 5% *w*/*v* of NaCl and 5% *w*/*v* of CaCl_2_ (pH 5.5) and a solution of HCl 1M (pH 0) were used at first. Then NaOH 4M (pH 14), HCl 4M (pH 0) and a concentrated salt mixture with both 10% *w*/*v* of NaCl and 10% *w*/*v* of CaCl_2_ (pH 5) were tested on the best performing samples of Table 1. Moving to a household/office environment, aggressive cleaning products such as a commercial basic degreaser (Chanteclair^®^) and a commercial acid detergent (Cillit Bang^®^) were tested. At last, food contact tests were performed using deionized water (MilliQ), acetic acid 3% *w*/*v*, ethanol 10% v/v and olive oil as food simulants named A, B, C and D respectively, according to UNI EN 1186-3:2002 and acetic acid 10 % *w*/*v* as a more aggressive test. Drop tests were also performed using real foods: Coca Cola^®^, sparkling white wine and hot coffee as produced by an Italian moka (approx. temperature 90° [25]).

### 5.3. Tests and Analysis Methods

#### 5.3.1. Drop Test

A direct, fast and very simple way of testing the resistance towards corrosion is the drop test. A drop of 30 μL of solution is deposed on the sample at time $t_{0}$, then after one hour ($t_{1}$), another drop of the same solution and volume is added on the previous drop and another drop is deposed on a new spot. At the end of the second hour $t_{2}$ two drops are added on those deposed at $t_{0}$ and $t_{1}$ and an extra third is deposed. When the test stops, usually after five hours (i.e., four drops) the drops are dried with paper towels and the samples are washed with deionized water and dried. In this way, the time effect can be demonstrated very easily, as can be seen in Figure 5. In the high temperature test, samples were put in a muffle at 50 °C for an hour. For this test, a 40 μL drop was used to compensate for evaporation. Since these analyses are qualitative, no replicates were carried out.

#### 5.3.2. Specific Migration Test

The migration in food simulants is determined using a procedure adapted from UNI EN 1186-1:2003 [26] and UNI EN 1186-3:2003 and the Guidelines on testing conditions for articles in contact with foodstuff [27] that are originally intended for testing plastics, as no specific regulation is available for magnesium alloys and all the used coatings are polymeric. The scope of the test in this case is to assess if the coating can prevent the migration of metals from the alloy. Each coated planar sample was immersed in deionized water (simulant A), or 3% acetic acid (simulant B) or 10% ethanol (simulant C), as showed in Figure 6. Polypropylene beakers with a diameter of 56 mm, height of 80 mm and capacity of 150 mL were used. The samples were immersed in 100 mL of simulant obtaining a surface/volume ratio of about 0.52. The tests were performed in different conditions ranging from 24 h at room temperature to 2 h at 70 °C.

#### 5.3.3. ICP-OES Analysis for Specific Migration Test

The analyses to determine the number of metals in the simulant solutions were carried out by emission spectroscopy by inductively coupled plasma (ICP-OES) using a Spectro Genesis ICP-OES spectrometer (Spectro Analytical Instruments, Kleve, Germany), equipped with a crossflow nebulizer and a Scott spray chamber. After the test, the recovered simulants were prepared for ICP-OES analysis adding 0.1 mL of HNO_3_ 69% to 1 mL of the solution and then MilliQ water to a volume of 10 mL. The elements were determined considering the spectral line providing the best signal/intensity ratio and on each solution three scans were performed and averaged. After the analysis of each solution the instrument performs a rinse cycle with 1% HNO_3_ solution to avoid contamination and memory effect between samples. The specific migration was calculated for each ICP-OES measure with three replicates. The data were multiplied by 10 to account for the dilution and scaled on the initial amount of simulant when evaporation was significant. The avg. of the measures on the reference was subtracted and the result (mg/L) divided by the exposed surface (dm^2^) to calculate the specific migration for each element. The three values for each measure were then averaged and the standard deviation calculated. The results are reported in the Supplementary Materials in Tables S1 and S2.

#### 5.3.4. ATR Analysis

The ATR analysis was performed on a Nicolet iN10 by Thermo Fischer Scientific (Waltham, MA, USA) with a Slide-On MicroTip Ge ATR crystal (throughput > 50%, 27° average angle). The detector was cooled with liquid nitrogen. The samples were tested after the drop test with the commercial products and after the aggressive drop test in the oven. A reference on the backside of the sample was first measured, followed by a measurement on the zone of the sample affected by the drop. Each time the background of air was taken before measuring the samples with an acquisition time of 51 s and a total number of acquisitions equal to 256.

#### 5.3.5. Electron and Optical Microscopy

A Hitachi FLEXSEM 1000 equipped with AZtecOne Oxford EDS was used for electron microscopy observation and EDS analysis. A tungsten filament was used as the electron source at 15 kV. The different coating materials were collected from the samples with a cutter and analyzed as such without coating (using low vacuum conditions to prevent samples from charging). Thickness was measured for those samples where a slice could be cut without fragmentation (PA11 and GSF). EDS analysis was performed on an average area of 0.010 mm^2^ to 0.025 mm^2^ on SE images. A STEMI 508 microscope with 2× frontal optics, Zeiss fiber optics halogen bulb and 20 MPx SONY sensor camera was used to collect optical images with high resolution.

## Figures and Tables

**Table 1:** Results of the food and soft chemicals tests for all tested samples.

Sample Name ^1^	Soft Chemical Aggression		Food Simulant Drop Test					Food Drop Test		
	**HCl 1M**	**Salt Mixture**	**MilliQ H_2_O**	**Acetic Ac. 3%**	**EtOH 10%**	**Oil**	**Acetic Ac. 10%**	**Coffee**	**Cola^®^**	**White Wine**
• Nanoc. Easysol	Fail	Fail	Pass	Fail	Pass	Pass	Fail	Fail	Fail	Fail
• Ceramic techsol	Fail	Pass	Pass	Fail	Pass	Pass	Fail	Pass	Fail	Fail
• Siliconic white	Pass	Pass	Pass	Pass	Pass	Pass	Pass	Pass	Pass	Pass
• Siliconic brown	Pass	Pass	Pass	Pass	Pass	Pass	Pass	Pass	Pass	Pass
• Lacquer GSF 12 μm	Fail	Fail	Pass	Pass	Pass	Pass	Pass	Pass	Pass	Pass
• Lacquer GSF 30 μm	Pass	Pass	Pass	Pass	Pass	Pass	Pass	Pass	Pass	Pass
• Lacquer GSF white 13 μm	-	-	Pass	Pass	Pass	Pass	Fail	Pass	Pass	Pass
• Lacquer GSF white 40 μm	Pass	Pass	Pass	Pass	Pass	Pass	Pass	Pass	Pass	Pass
• PA11 transparent	Pass	Pass	Pass	Pass	Pass	Pass	Pass	Pass	Pass	Pass
• PA11 white	Pass	Pass	Pass	Pass	Pass	Pass	Pass	Pass	Pass	Pass

^1^ For a faster recognition of the class of each sample, a colored dot was added to the names of the samples in all the tables. A pale brown dot (•) indicates that the sample has a silicon-based coating. A black dot (•) indicates that the sample has an organic-based coating. A green dot (•) indicates that the sample has a biobased coating. The same color code apply to Table 2 and Table 3.

**Table 2:** Results of harsh chemicals tests for samples passing the first level of tests.

Sample Name	Hard Chemical Aggression			Household Drop Test	
	**HCl 4M**	**NaOH 4M**	**Conc. Salt Mixture**	**Chanteclair^®^**	**Chillit Bang^®^**
• Siliconic white	Pass	Fail	Pass	Pass	Pass
• Siliconic brown	Pass	Fail	Pass	Pass	Pass
• Lacquer GSF 30 μm	Fail	Fail	Fail	Fail	Fail
• Lacquer GSF white 40 μm	Pass	Pass	Pass	Pass	Pass
• PA11 transparent	Pass	Pass	Pass	Pass	Pass
• PA11 white	Pass	Pass	Pass	Pass	Pass

**Table 3:** Results of the migration tests for the more promising coatings.

Sample Name	Migration Test and Coffee Test	Migration Test on Prototypes	
	**Acetic Ac. 3% on Flat Samples**	**Simulants A B C in Cups**	**Coffee**
• Siliconic white	Fail	Fail	Fail
• Siliconic brown	Fail	-	-
• Lacquer GSF white 40 μm	Pass	Pass	Pass
• PA11 transparent	Pass	Pass	Pass
• PA11 white	Pass	Pass	Pass

## Data Availability

Not applicable.

## Supplementary Materials

The file containing the data about specific migration is available online at https://www.mdpi.com/article/10.3390/ijms22094915/s1; the description of the file is available in Section 5.3.3.
