# Supplementary material for: Low-Cost Biobased Coatings for AM60 Magnesium Alloys for Food Contact and Harsh Environment Applications
Source: Int J Mol Sci. 2021 May 6;22(9):4915. doi: 10.3390/ijms22094915 (PMC8124156; doi:10.3390/ijms22094915)
Supplement: Supplementary file 1 [file ijms-22-04915-s001.zip › ijms-1179561-supplementary.pdf]

Table S1: Specific migration values of selected metals. Test performed on flat samples with simulant B (acetic acid 3%) at room temperature.

	Al	Co	Cr	Cu	Fe	Mg	Mn	Ni	S	Ti	V	Zn	As	Ba	Cd	Mo	Pb
	mg/dm^2	mg/dm^2	mg/dm^2	mg/dm^2	mg/dm^2	mg/dm^2	mg/dm^2	mg/dm^2	mg/dm^2	mg/dm^2	mg/dm^2	mg/dm^2	mg/dm^2	mg/dm^2	mg/dm^2	mg/dm^2	mg/dm^2
Simulant B 24h RT PA 11 transparent	<LOD	<LOD	<LOD	<LOD	<LOD	<LOD	<LOD	<LOD	<LOD	<LOD	<LOD	<LOD	<LOD	<LOD	<LOD	<LOD	<LOD
Simulant B 24h RT siliconic brown	0.016 ± 0.002	<LOD	<LOD	<LOD	0.0013 ± 0.0006	1.24 ± 0.07	0.0004 ± 0.0002	<LOD	<LOD	<LOD	<LOD	<LOD	<LOD	<LOD	<LOD	<LOD	<LOD
Simulant B 2h RT GSF lacquer transparent	13 ± 2	<LOD	<LOD	<LOD	0.0007 ± 0.0009	231 ± 26	0.45 ± 0.05	0.0012 ± 0.0007	<LOD	<LOD	<LOD	<LOD	<LOD	0.0007 ± 0.0003	<LOD	<LOD	<LOD
LOD of ICP-OES (mg/l)	0.003	0.002	0.2	0.02	0.01	0.01	0.00022	0.008	0.06	0.002	0.002	0.01	0.01	0.0001	0.002	0.009	0.02

Table S2: Specific migration values of selected metals. Test performed on prototype cups.

		Al	Cu	Mg	Mn	Ni	As	Cd	Mo	Pb
		mg/dm^2	mg/dm^2	mg/dm^2	mg/dm^2	mg/dm^2	mg/dm^2	mg/dm^2	mg/dm^2	mg/dm^2
Simulant A 70°C for 2 hours	PA transparent	<LOD	<LOD	<LOD	<LOD	0.0006 ± 0.0005	<LOD	<LOD	<LOD	<LOD
	PA transparent (sanded)	<LOD	<LOD	<LOD	<LOD	0.0016 ± 0.0008	<LOD	<LOD	<LOD	<LOD
	PA white	<LOD	<LOD	<LOD	<LOD	0.0018 ± 0.0009	<LOD	<LOD	<LOD	<LOD
Simulant A 20°C for 24 hours	PA transparent	0.0022 ± 0.0004	<LOD	<LOD	<LOD	<LOD	<LOD	<LOD	<LOD	<LOD
	PA transparent (sanded)	<LOD	<LOD	<LOD	<LOD	<LOD	<LOD	<LOD	<LOD	<LOD
	PA white	<LOD	<LOD	<LOD	<LOD	<LOD	<LOD	0.0008 ± 0.0003	<LOD	<LOD
Simulant C 20°C for 24 hours	PA transparent	<LOD	<LOD	<LOD	<LOD	<LOD	<LOD	<LOD	0.0013 ± 0.0008	0.005 ± 0.002
	PA transparent (sanded)	<LOD	0.0020 ± 0.0004	<LOD	<LOD	0.0014 ± 0.0001	<LOD	<LOD	0.0013 ± 0.0008	0.006 ± 0.003
	PA white	<LOD	<LOD	<LOD	<LOD	<LOD	<LOD	<LOD	0.006 ± 0.002	0.027 ± 0.007
Simulant B 70°C for 2 hours	Laquer GSF white 20um	<LOD	<LOD	0.095 ± 0.002	0.00042 ± 0.00002	<LOD	<LOD	<LOD	<LOD	<LOD
	Siliconic white	0.0175 ± 0.0002	<LOD	0.702 ± 0.005	0.00061 ± 0.00003	<LOD	<LOD	<LOD	<LOD	<LOD
	PA transparent	<LOD	<LOD	<LOD	<LOD	0.00021 ± 0.00008	<LOD	0.0002 ± 0.0001	<LOD	<LOD
	PA transparent (sanded)	<LOD	<LOD	<LOD	0.0002 ± 0.0002	<LOD	<LOD	<LOD	<LOD	<LOD
	PA white	<LOD	<LOD	<LOD	<LOD	<LOD	<LOD	<LOD	<LOD	<LOD
Simulant B 20°C for 24 hours	PA transparent	<LOD	<LOD	<LOD	<LOD	<LOD	<LOD	<LOD	<LOD	<LOD
	PA transparent (sanded)	<LOD	<LOD	<LOD	<LOD	0.0003 ± 0.0002	<LOD	<LOD	<LOD	<LOD
	PA white	<LOD	<LOD	<LOD	<LOD	<LOD	<LOD	<LOD	<LOD	<LOD

Table S3: Specific migration limits for metals from the Regulation (EU) 2017/752 amending Regulation (EU) 10/2011 (Table 1 Annex II)

Metal	Limit (mg/kg)
Al	1
Ba	1
Co	0.05
Cr	ND (LOD <0.01)
Cu	5
Fe	48
Mn	0.6
Ni	0.02
Zn	5
Mo	0.12
V	0.01
As	0.002
Cd	ND (LOD < 0.002)
Pb	ND (LOD <0.01)
Mg	-

*The conversion of the data in mg/dm^2 to mg/kg to compare the obtained specific migrations with migration limits can be made according to art.17 of the (UE) N. 10/2011 that states that for containers and other articles, containing or intended to contain, less than 500 millilitres or grams the value of migration shall be expressed in mg/kg applying a surface to volume ratio of 6 dm^2 per kg of food.

Table S4: IR bands assignment

Sample	cm^{-1}	Assignment
Nanoceramic	1092	Si-O-Si asymm. Str.
	1423	Si-CH_3 deform.
Siliconic	844	Si-OH str.
	1050 - 1200	Si-O-Si asymm. Str.
	1228	Si-CH_2R
GSF laquer	1720	C=O str.
	1234	C-O-C asymm str.
	2935 - 2854	CH str.
	3350	OH str. Of TiO_2 in the white sample
PA 11	3305	NH str.
	1543	NH bend.
	2920 , 2850	CH_2 symm. Str.
	1635	C=O str.
	1469	CH_2 bend.
	717	CH_2 rocking

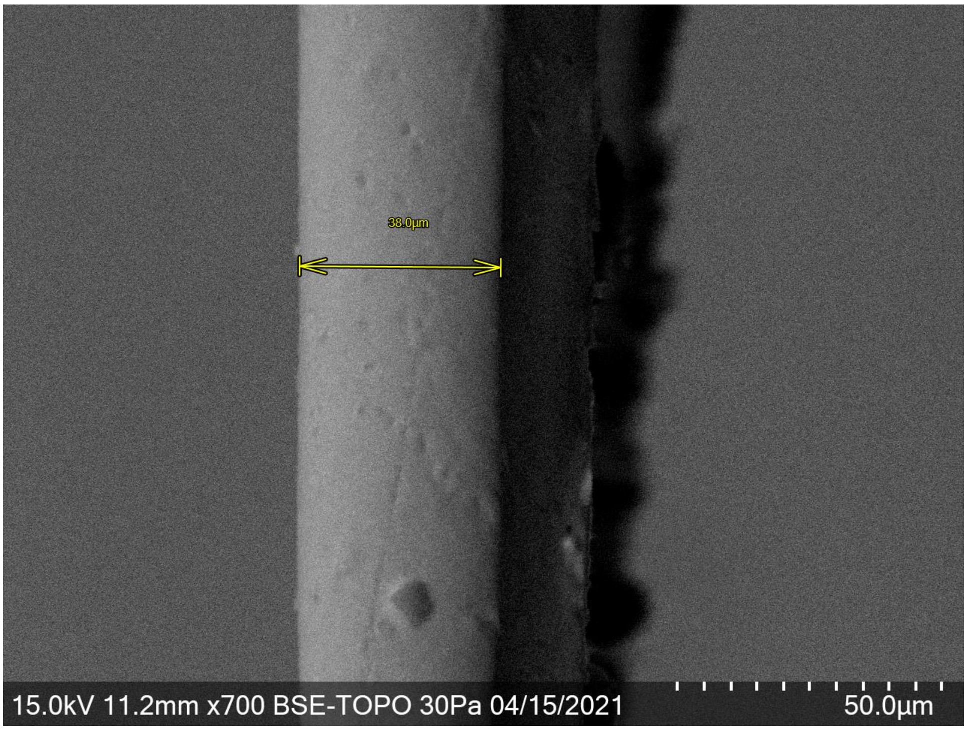

Figure S1: SEM image of the cross section of the slice cut from GSF lacquer 40 μm.
